# Preliminary evidence for the validity of the Brief Post-Secondary Student Stressors Index (Brief-PSSI): A cross-sectional psychometric assessment

**DOI:** 10.1371/journal.pone.0297171

**Published:** 2024-01-19

**Authors:** Brooke Linden, Amy Ecclestone

**Affiliations:** 1 Health Services and Policy Research Institute, Queen’s University, Kingston, Ontario, Canada; 2 School of Public Health Sciences, University of Waterloo, Kitchener, Ontario, Canada; The John Paul II Catholic University of Lublin, POLAND

## Abstract

The brief version of the Post-Secondary Student Stressors Index (Brief-PSSI) was developed in order to improve the usability of the instrument as a method for evaluating the severity and frequency of stressors faced by post-secondary students. While the original 46-item instrument has been thoroughly psychometrically validated and successfully used among student populations, the length of the instrument limits its utility. Providing a valid, shortened version of the PSSI will enable institutions to include the tool on existing online surveys currently being deployed to surveil the mental health and wellbeing of their students. This study reports preliminary evidence in support of the validity and reliability of the Brief-PSSI using a cross-sectional pilot sample of students attending an Ontario university in 2022. A total of 349 participants (average age 25 (*SD* = 7.7), range 19–60) completed the first survey, while 149 completed the follow-up survey (average age of 26 (*SD* = 7.7), range 17–60). Evidence of internal structure, relations to other variables, and of test-retest reliability was assessed according to established index validation guidelines, including the specification of multiple-indicator, multiple-cause models, and Spearman’s rho correlation coefficients. Results provide preliminary support for the validity and reliability of the tool, which demonstrated acceptable goodness-of-fit statistics, statistically significant relationships with like constructs in the hypothesized directions, and good test-retest reliability correlation coefficients. The Brief-PSSI is a useful tool for evaluating the sources of stress among post-secondary students, assessing both the severity of stress experienced and frequency with which each stressor occurred. Future research should explore the practical utility of adding the Brief-PSSI to existing survey assessments as well as pursue the continued collection of validation evidence for the tool among varied student populations.

## Background

Concerns surrounding post-secondary student mental health and wellbeing have increased over the past decade, with above average stress levels and related mental health concerns reported by many in this population [1, 2]. The majority of students attending post-secondary institutions in Canada belong to the 18-to-25 year age group, referred to as “emerging adulthood” [3]. Research suggests that during this phase of the life course, increased autonomy combined with a lack of role permanence and associated responsibilities results in an increased propensity to engage with risky behaviour, often as maladaptive methods of coping with stress [3]. Importantly, substantial brain and psychosocial development take place during emerging adulthood, placing individuals at increased susceptibility to risk factors for the development of mental illnesses [4]. In fact, the majority of mental illnesses have their first onset on or before 18 years of age [5]. Data collected from the National College Health Assessment survey (NCHA II) in 2019 (*n* = 55,284) supports this, with nearly one quarter of respondents self-reporting a past-year diagnosis of anxiety (24%) or depression (20%), with 16% reporting a dual-diagnosis [6]. In addition, many students reported feeling hopeless (63.6%), overwhelmed (88.2%) and anxious (68.9%) (6). Concerningly, recent research suggests that these prevalence estimates have increased significantly over the past decade [7].

In addition to diagnoses and symptoms of mental illnesses, a high prevalence of above average stress was evident in students’ responses to the NCHA II. Nearly half of respondents reported their past-year stress level to have been “more than average” (45.6%), while 15% reported “tremendous” stress [6]. Excessive stress among student populations has been linked to declining mental health, which is in turn associated with a number of negative outcomes including the development of mental illnesses [8, 9], poor academic performance [10], increased substance use [1], dropout [11], burnout [12], and in extreme cases, self-injury and/or suicidal ideation [2, 9]. Though much of the extant literature highlighting student stress focuses on stressors related to academics, more contemporary research suggests that the stressors experienced by students throughout their post-secondary careers extend into additional domains including the learning environment, campus culture, interpersonal, and personal [13]. More recently, challenges related to the COVID-19 pandemic introduced novel stressors (and in some cases, exacerbated pre-existing ones) including a mandatory move to online learning, interrupted academic activities and work placements, increased social isolation and loneliness stemming from campus closures, and economic uncertainty [14, 15].

The Post-Secondary Student Stressors Index (PSSI) was developed in collaboration with students, aiming to fill the gaps left by previous instruments that attempted to assess students stress [16–18]. These instruments were either too narrow or broad in scope [19], did not involve students in the development process [20], had poor psychometric properties [21–24], or were outdated [20, 25]. Most importantly, they did not comprehensively capture the multitude of stressors faced by post-secondary students. The PSSI is a validated tool that evaluates 46 stressors pertinent to post-secondary life across five domains—academics, the learning environment, campus culture, interpersonal and personal stressors—the PSSI can help post-secondary institutions to determine the most severe and frequently occurring sources of stress for students on their campus and tailor their upstream mental health supports accordingly. Although the PSSI is a psychometrically sound and evidence-informed tool, its length may be problematic for inclusion on pre-existing surveillance survey efforts currently conducted by institutions (i.e., the National College Health Assessment, Canadian Campus Wellbeing Survey), with 6 to 12 items in each assessed domain. Therefore, the purpose of this study was to collect and analyze preliminary evidence for the validity and reliability of the Brief-PSSI, a shorter, 14-item version of the original PSSI.

## Methods

### Study design

We used a cross-sectional, online survey to gather data from a random sample of students attending an Eastern Ontario University during the Spring semester of 2022. All enrolled students were eligible for inclusion with the exception of those studying off campus (i.e., on exchange abroad). Upon request, a sample of 5000 student e-mails was drawn by Queen’s University’s Office of Research and Institutional Planning and provided to the Principal Investigator. The sample size of 5000 was requested based on our expectation of obtaining an approximate response rate of 8–10% based on previous experience surveying this population. We aimed to obtain a minimum of 300 responses in order to comfortable conduct the desired psychometric analyses of interest.

Students received an e-mail invitation on July 5, 2022, with a reminder email sent to those who had not yet completed the survey on July 12, 2022. Participants were provided with a Letter of Information as the first page of the survey and were asked to indicate their written, informed consent by selecting “I have read the Letter of Information and agree to participate in this study” before being granted access to any survey questions. To enable us to assess the test-retest reliability of the tool, a second, follow-up survey was sent to all participants who completed the first survey on August 4, 2022, with a reminder e-mail sent on August 12, 2022.

This study sought to obtain preliminary evidence for the validity and reliability of the Brief-PSSI. *Validity* is a process by which we determine the degree of confidence we can place on the inferences made about people based on their scores on a given instrument [26]. *Reliability* refers to the consistency of test scores within a particular population. As stated in the *Standards for Educational and Psychological Testing* (“the *Standards*”), the comprehensive validation of an instrument requires the accumulation of evidence from five sources: content; response processes; internal structure; relations to other variables; and test consequences [27]. This article reports the collection of internal structure and relations to other variables evidence for the validity of the Brief-PSSI, in addition to examining its test-retest reliability. This research received ethics clearance from Health Sciences and Affiliated Teaching Hospitals Research Ethics Board (#HSPR-020-22).

### Measures

#### Demographics

Several demographic variables were assessed, including gender (male, female, non-binary), year of birth, residence during the academic year, level of study (undergraduate, graduate, or professional program), enrollment status (part-time/full-time), international student status (yes/no), first generation student status (yes/no), racialized group/visible minority status (yes/no), and self-reported grade point average (GPA).

#### Brief Post-Secondary Student Stressors Index

The Brief-PSSI is composed of 14 items and was developed by strategically collapsing categories of items included in the original 46-item version of the PSSI. For each stressor on the Brief-PSSI, respondents are asked to indicate the severity of stress experienced and the frequency with which this stress occurred. Response options range on a scale from 1 (‘not stressful’ and ‘rarely’) to 4 (‘very stressful’ and ‘almost always’), with higher ratings indicating a greater severity or frequency of stress. An additional option to indicate ‘N/A’ was also available in cases where a stressor did not occur or was not applicable.

#### Mental health measures

Composed of ten items, the Perceived Stress Scale (PSS-10) is designed to evaluate overall perceived stress level. Items ask respondents how often they felt a certain way within the past month, with response options ranging from 0 (*never*) to 4 (*very often*). A composite score ranging from 0 to 40 is calculated, with higher scores being indicative of higher stress levels [28]. The Kessler Psychological Distress Scale (K10) measures levels of psychological distress experienced within the past month. Each of the ten items are scored from 1 (*none of the* time) to 5 (*all of the* time). A composite score ranging from 10 to 50 is calculated, with higher scores being indicative of higher levels of psychological distress [29]. Finally, the Connor-Davidson Resiliency Scale (CD-RISC-10) assesses an individual’s ability to cope in the face of adversity. Items are scored from 0 (*not true at all*) to 4 (*true nearly all of the time*). Responses are summed for a composite score ranging from 0–40, with higher scores indicative of a higher level of resilience [30, 31]. All three scales have demonstrated strong psychometric properties and have been used among samples of post-secondary aged youth [32–36].

### Analysis

Statistical analyses were completed using R (R Foundation for Statistical Computing, Vienna, Australia) Version 4.1.3 [37]. We first conducted a basic descriptive analysis on the demographic characteristics of the sample and each of the stressors variables in the Brief-PSSI. Following this, we began our psychometric assessment of the instrument. We used a statistical approach to assessing evidence for validity appropriate for an index, as opposed to a scale. Like the original PSSI, the brief version of the tool was designed as an index, meaning that individual stressors were conceptualized as causal indicators (e.g., “causes” of stress), rather than effect indicators (e.g., “effects” of stress) [38, 39]. Index construction places emphasis on considering multicollinearity among indicators and assessing theoretical relationships between latent constructs and predictor variables [40] rather than homogeneity of items within an index [39, 41].

#### Item reduction

An exploratory factor analysis (EFA) was performed on the original, 46-item PSSI to guide the item reduction process. The results of the EFA were examined for clear groupings of factor loadings. Groupings were then examined for a common theme and “collapsed” into a single item for use on the Brief PSSI. For example, original items “maintaining my GPA” and “receiving a bad grade” were collapsed to create a new item for the brief tool, “maintaining my grades”. Where cross-loadings occurred (i.e., factor loadings on more than one component), the higher factor loading was retained. See S2 Appendix for the complete results of the EFA and resulting item reduction.

#### Internal structure evidence

First, we performed an EFA on the Brief-PSSI to determine the recommended structure of the instrument (S3 Appendix). Both parallel and very simple structure analyses recommended a one-factor solution as the best fit, closely followed by a two-factor solution. We elected to test both models in subsequent analyses. Next, to determine the degree to which the relationships among items within the instrument were consistent with what is expected of the construct under study, we estimated multiple-indicator, multiple-cause (MIMIC) models, a special case of structural equation modelling (SEM) and extension of Confirmatory Factor Analysis (CFA). MIMIC models allow for the estimation of relationships among latent constructs of interest (unobserved variables such as stress) [42]. We hypothesized the model would demonstrate acceptable goodness-of-fit statistics with no evidence of multicollinearity among indicators (using a variance inflation factor (VIF) cut-off threshold of 10) [43].

#### Relations to other variables

To explore the relationships between the Brief-PSSI and other “like” constructs, we calculated non-parametric Spearman’s rho correlation coefficients to further examine relationships between items on the Brief-PSSI and like constructs. We hypothesized that a higher score on the Brief-PSSI latent variable (indicating higher student stress) would predict higher scores on both the PSS-10 and K10 (higher general stress and psychological distress) and lower scores on the CD-RISC-10 (lower resilience).

#### Reliability

To assess the test-retest reliability of the tool, we used the matched responses from both administered surveys to examine Spearman’s rho correlation coefficients between items on the Brief-PSSI over the two-week period. We hypothesized that student stress levels would remain fairly consistent over a two-week period (barring any major stressful event during that time frame) and considered 0.7 to be indicative of good test-retest reliability [26]. We then conducted a sensitivity analysis, removing participants who indicated they had experienced a major stressful event during the two-week period between surveys. Tests were conducted using a complete case analysis approach to missing data, wherein any respondent with complete information for the variables used in an individual test was included in the analyses.

## Results

### Sample and participants

We first calculated the descriptive statistics for the sample (Table 1). As expected, we initially received 506 submitted surveys representing a 10% response rate, but only *n* = 349 had usable data (i.e., <90% missing data) ultimately resulting in a response rate closer to 7%. A total of 174 of these participants completed the second survey (T2 *n* = 174), representing an approximate 50% follow-up response rate between T1 and T2. At both time points, the median age was 23 years of age, and most participants were full-time, female undergraduate students between 21 and 24 years old, reporting a GPA in the A range. For the majority of our analyses, we used the T1 sample as it was larger. We used both samples to evaluate the test-retest reliability of the tool.

**Table 1:** Demographic characteristics of samples.

	T1 (*n* = 349)	T2 (*n* = 174)
	n (%)	n (%)
**Gender**		
Male	79 (22.6)	40 (21.4)
Female	248 (71.1)	131 (70.1)
Non-binary	13 (3.7)	9 (4.8)
Prefer not to self-describe	7 (2.0)	4 (2.1)
Prefer not to answer	2 (0.6)	3 (1.6)
**Age**		
20 years and under	91 (26.1)	46 (25.0)
21–24 years	123 (35.2)	63 (34.2)
25–29 years	67 (19.2)	36 (19.6)
30 years and over	68 (19.5)	39 (21.2)
**Self-identification as a racialized person or member of visible minority**		
Yes	108 (30.9)	54 (28.9)
No	232 (66.5)	128 (68.4)
Prefer not to answer	9 (2.6)	5 (2.7)
**Residence during the school year**		
On campus, in residence	46 (13.2)	22 (11.8)
Other on campus housing	9 (2.6)	5 (2.7)
Off campus with housemates	147 (42.1)	84 (44.9)
Off campus alone	56 (16.0)	28 (15.0)
Off campus with family	87 (24.9)	47 (25.1)
Prefer not to answer	4 (1.1)	1 (0.5)
**Level of study**		
Undergraduate	200 (57.3)	104 (55.6)
Master’s level degree	92 (26.4)	54 (28.9)
Doctoral level degree	41 (11.7)	23 (12.3)
Professional program	12 (3.4)	5 (2.7)
Other	4 (1.1)	0 (0.0)
Prefer not to answer	0 (0.0)	1 (0.5)
**Enrollment status**		
Full-time student	315 (90.3)	171 (91.4)
Part-time student	30 (8.6)	16 (8.6)
Other	4 (1.1)	0 (0.0)
Prefer not to answer	0 (0.0)	0 (0.0)
**International student**		
Yes	36 (10.3)	21 (11.2)
No	311 (89.1)	165 (88.2)
Prefer not to answer	2 (0.6)	1 (0.5)
**First-generation student**		
Yes	65 (18.6)	30 (16.0)
No	283 (81.1)	156 (83.4)
Prefer not to answer	1 (0.3)	1 (0.5)
**Faculty of study**		
Faculty of Arts and Science	147 (42.1)	81 (43.3)
Faculty of Education	35 (10.0)	15 (8.0)
Faculty of Engineering and Applied Science	54 (15.5)	34 (18.2)
Faculty of Health Science	80 (22.9)	42 (22.5)
Faculty of Law	1 (0.3)	1 (0.5)
School of Business	31 (8.9)	14 (7.5)
School of Policy Studies	1 (0.3)	0 (0.0)
**Self-reported grade point average (GPA)**		
A (80–100%)	244 (69.9)	138 (73.8)
B (70–70%)	77 (22.1)	36 (19.3)
C (60–69%)	21 (6.0)	9 (4.8)
D (50–59%)	1 (0.3)	1 (0.5)
F (49% and below)	0 (0.0)	0 (0.0)
Prefer not to answer	6 (1.7)	3 (1.6)

Note: Responses of “prefer not to answer” were coded as missing for subsequent analyses.

### Distribution of stressors in sample

**Fig 1:**
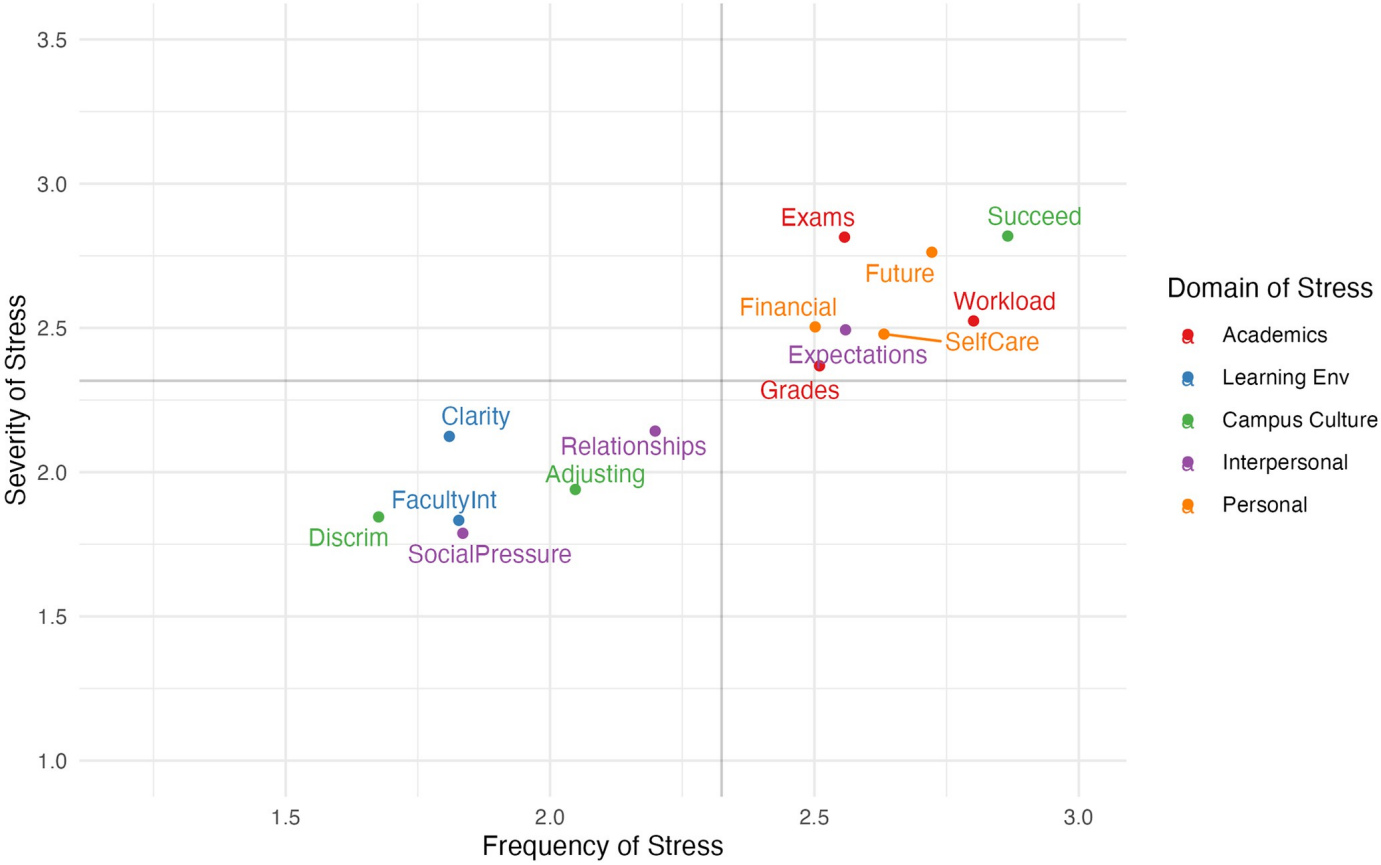
Quadrant graph displaying mean severity and frequency of Brief-PSSI stressors. Stressors are displayed by mean severity and frequency ratings for the total sample, colour coded by domain (T1 data).

**Fig 2:**
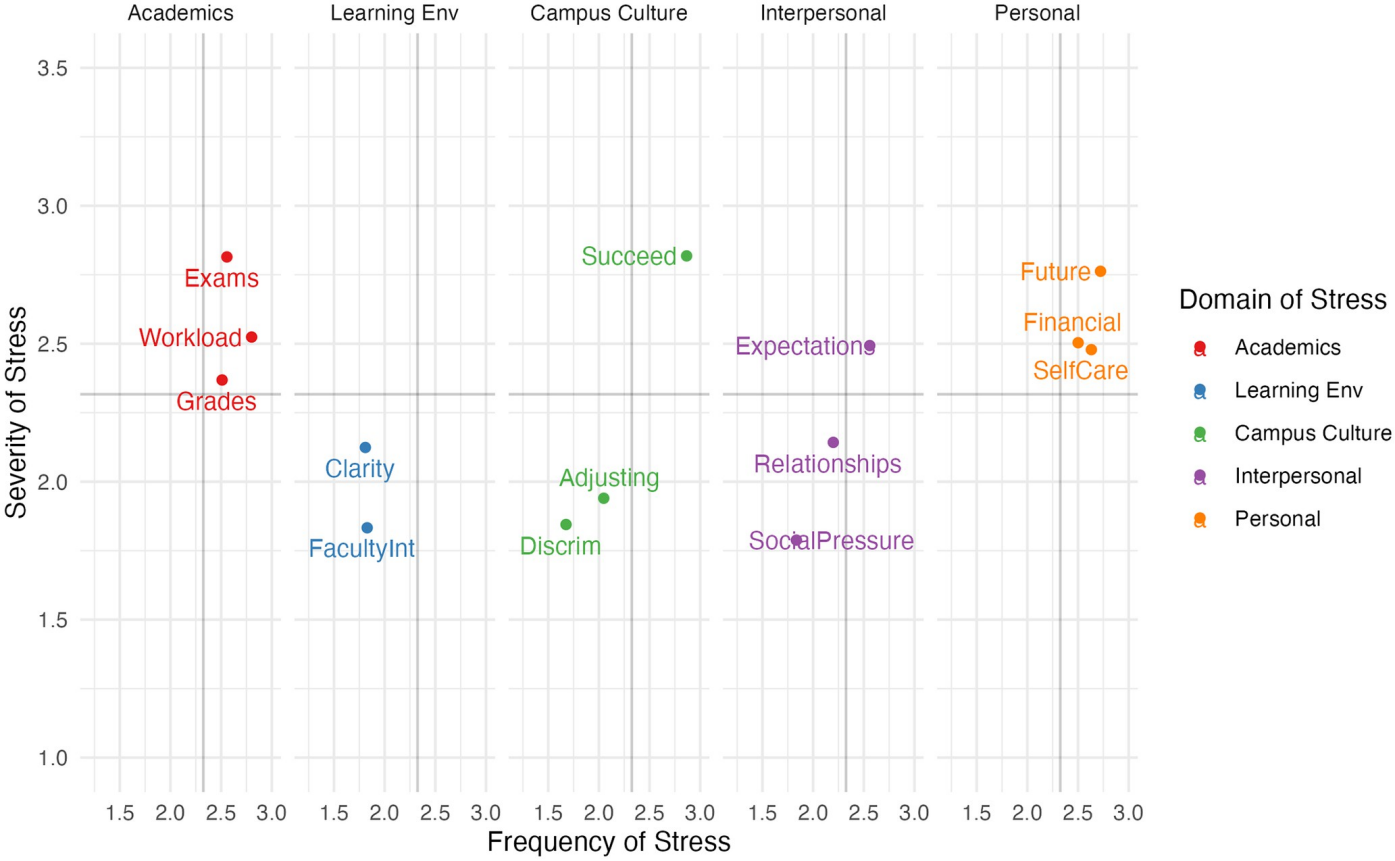
Facet graph displaying mean severity and frequency of Brief-PSSI stressors. Stressors are displayed by mean severity and frequency ratings for the total sample, faceted and colour coded by domain. Note that the same data is displayed in Figs 1 and 2 (T1 data).

Descriptive statistics (means and standard deviations) were calculated for each stressor on the Brief-PSSI at each timepoint (see S1 Appendix). The overall distribution of stressors, stratified by mean severity and frequency for the T1 sample is depicted via a quadrant graph in Fig 1. Fig 2 depicts the same data in a faceted display, where each domain of stress is shown on its own quadrant graph. The most severe stressors experienced by students in this sample were *pressure to succeed* ($\bar{X}=2.89$, SD = 0.94), *examinations* ($\bar{X}=2.84$, SD = 0.85), and *concerns for the future* ($\bar{X}=2.83$, SD = 1.03). The most frequent stressors identified were *pressure to succeed* ($\bar{X}=2.84$, SD = 0.97), *managing my academic workload* ($\bar{X}=2.78$, SD = 0.88), and *concerns for the future* ($\bar{X}=2.71$, SD = 1.05). Overall, these findings are consistent with patterns of stressors we have observed in previous samples of students who completed the original PSSI [13, 16, 17].

### Internal structure

Using data from the first time point, we first performed two exploratory factor analyses based on the recommended results of parallel and very simple structure analyses: a one-factor solution, and a two-factor solution. The one-factor model was significant (*X*^*2*^ = 312.5, *p*<0.001), with most factor loadings ≥0.4, 29.7% of the variance explained, and acceptable fit statistics (RMSA = 0.07, RMSEA = 0.094 [95% CI 0.083, 0.105] and TLI = 0.79). The two-factor model was also significant (*X*^*2*^ = 208.19, *p*<0.001), with factor loadings mostly ≤0.4, and 35.1% of the total variance explained. The fit for this model was good (RMSA = 0.05, RMSEA = 0.08 [95% CI 0.68, 0.093] and TLI = 0.84). The table in S3 Appendix displays the EFA results.

Next, we specified two MIMIC models. We first ruled out issues of multicollinearity by confirming that all variance inflation factors (VIF) for all Brief-PSSI indicators were well below the selected cut-off (<5). Descriptive statistics for indicators included in the models are outlined in Table 2. In the first model, we included all 14 stressors variables from the Brief-PSSI, specifying one overall latent variable of “student stress”. In the second model, we ran the two-factor model we explored earlier through EFA. For both models, we excluded observations where a participant indicated that a stressor was “not applicable” or “did not happen” based on prior analyses that demonstrated the removal of these responses made for a stronger overall model [18]. Results of both models are displayed in Table 3.

**Table 2:** Descriptive statistics for MIMIC model indicators.

	Mean	SD	% NA
**Indicators**			
Examinations (i.e., midterms, finals)	2.84	0.85	1.58
Managing my academic workload	2.54	0.77	0.00
Managing my grades	2.41	0.93	4.58
Lack of clarity in course instruction	2.12	0.94	19.77
Interacting with faculty	1.82	0.85	2.29
Adjusting to university life	1.97	0.95	11.75
Pressure to succeed	2.89	0.94	0.57
Discrimination (e.g., racism, sexism, etc.)	1.83	0.83	38.40
Managing relationships	2.18	0.90	5.16
Social pressures (e.g., drinking, going out late, putting socializing before schoolwork)	1.83	0.92	18.62
Meeting performance expectations	2.55	0.91	1.15
Managing self-care and health (e.g., nutrition, exercise, taking time to rest or engage with hobbies)	2.51	1.00	1.43
Financial concerns	2.53	1.05	8.88
Concerns for the future (e.g., finding employment after graduation, hitting lifetime milestones)	2.83	1.03	3.72

Notes: Means were calculated excluding responses of 0 (“Didn’t Happen” or “Not Applicable”); SD = Standard Deviation; % NA = Proportion of missing responses. Abbreviations: Perceived Stress Scale 10-item version (PSS-10), Kessler Psychological Distress Scale 10-item version (K10), Connor Davidson Resiliency Scale 10-item version (CD-RISC-10).

**Table 3:** Results of multiple-indicator, multiple-cause (MIMIC) models.

Parameter	Indicators	Model 1 (One-factor)		Model 2 (Two-factor)	
		(n = 128)		(n = 128)	
		*Factor*	*Estimates*	*Factor*	*Estimates*
*Factor Loading*	Examinations (i.e., midterms, finals)	Student Stressors	0.631	Academic	0.641
	Managing my academic workload		0.708		0.729
	Managing my grades		0.714		0.769
	Pressure to succeed		0.697		0.685
	Meeting performance expectations		0.755		0.780
	Concerns for the future (e.g., finding employment after graduation, hitting lifetime milestones)		0.572	Other Stressors	0.580
	Lack of clarity in course instruction		0.431		0.476
	Interacting with faculty		0.302		0.331
	Adjusting to university life		0.573		0.617
	Discrimination (i.e., racism, sexism, etc.)		0.383		0.401
	Managing relationships		0.472		0.543
	Social pressures (e.g., drinking, going out late, putting socializing before schoolwork)		0.471		0.540
	Managing self-care and health (e.g., nutrition, exercise, taking time to rest or engage with hobbies)		0.568		0.616
	Financial concerns		0.411		0.431
*Fit Statistics*	X2		143.47***		122.66**
	TLI		0.847		0.901
	RMSEA (95% CI)		0.08 (0.06, 0.10)		0.07 (0.05, 0.09)
	SRMR		0.07		0.06

Abbreviations: Perceived Stress Scale 10-item version (PSS-10), Kessler Psychological Distress Scale 10-item version (K10), Connor Davidson Resiliency Scale 10-item version (CD-RISC-10).

All items in the first model demonstrated moderate-to-strong factor loadings, with only two falling <0.4. The model was statistically significant (*X*^*2*^ = 143.47, *p*<0.001) and demonstrated acceptable overall fit. The root mean square error of approximation (RMSEA) and standardized root mean square residual (SRMR) were both <1, though not as small as typically desired (≤0.05), while the Tucker-Lewis Index (TLI) was just below the desired 0.9 cutoff. The second model was also statistically significant (*X*^*2*^ = 122.66, *p*<0.001) and showed slightly stronger fit statistics. RMSEA and SRMR were smaller compared to the first model, while the TLI reached the desired 0.9 cutoff.

### Relations to other variables

**Fig 3:**
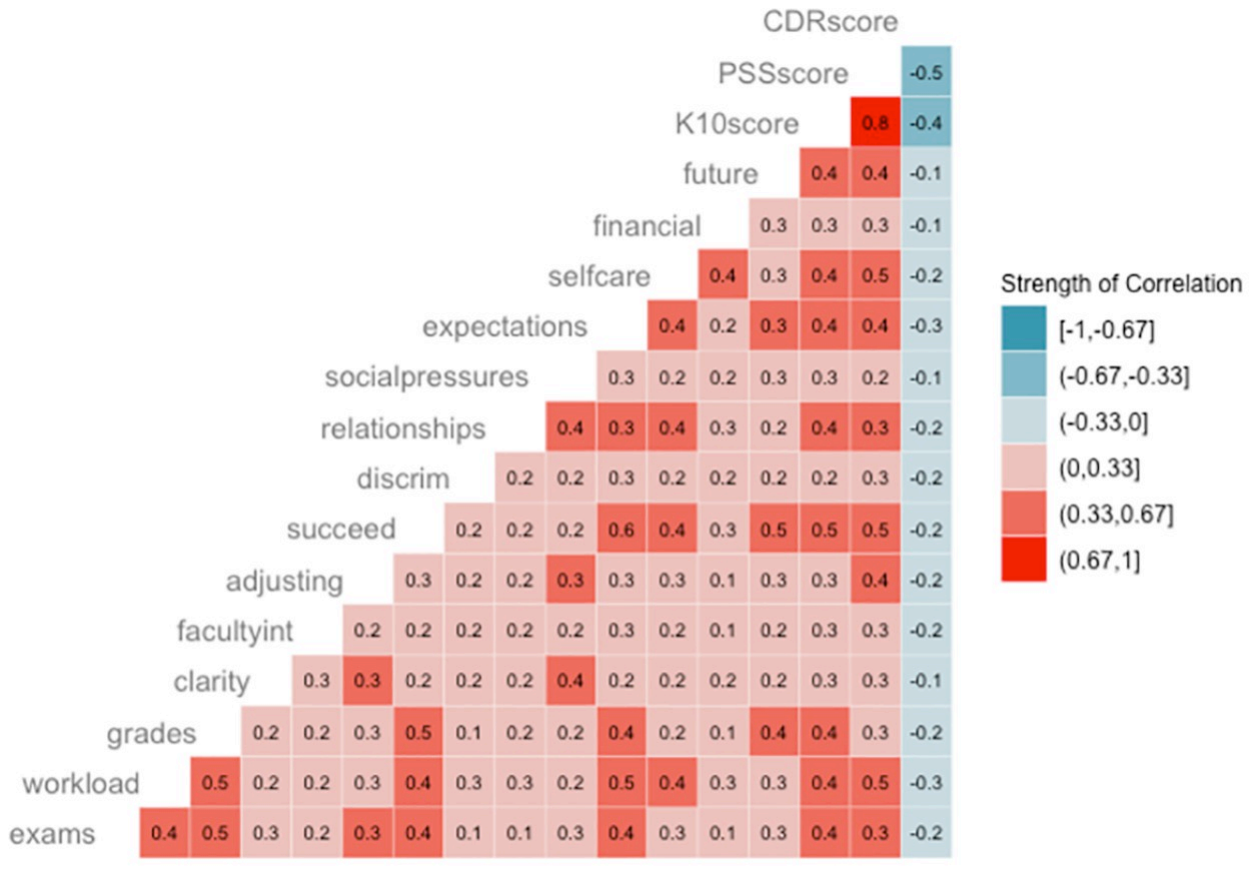
Correlation matrix for Brief-PSSI stressors and related constructs. Data shows the relationships between individual stressors and the three main related constructs of interest: the Kessler Psychological Distress Scale (K10, “CDRscore”), Perceived Stress Scale (PSS10, “PSSscore”), and Connor-Davidson Resiliency Scale (CD-RISC, “CDRscore”).

To assess relations to other variables, the 14 items on the Brief-PSSI were correlated with the PSS-10, K10, and CD-RISC-10 scores. As expected, all correlations moved in the hypothesized direction (i.e., positively related to PSS-10 and K10, inversely related to CD-RISC-10) and were statistically significant (Fig 3).

### Test-retest reliability

Next, we assessed the temporal stability of the tool by examining Spearman’s rho (*r*_*s*_) correlation coefficients between Brief-PSSI items and PSS-10 scores at the first and second data collection time points for all respondents with complete data (i.e., who answered the questions on both surveys). As hypothesized, all correlations were moderate-to-strong (*r*_*s*_ = 0.50–0.73) and statistically significant (Table 4).

**Table 4:** Test-retest reliability correlation coefficients for the Brief-PSSI and PSS-10 at T1 and T2.

	r_s_	95% CI	n
**Brief-PSSI Items**			
Examinations (i.e., midterms, finals)	0.72	0.63, 0.79	142
Managing my academic workload	0.52	0.41, 0.63	172
Managing my grades	0.73	0.65, 0.80	166
Lack of clarity in course instruction	0.50	0.35, 0.61	132
Interacting with faculty	0.61	0.51, 0.70	159
Adjusting to university life	0.66	0.55, 0.74	143
Pressure to succeed	0.63	0.53, 0.71	171
Discrimination (i.e., racism, sexism, etc.)	0.65	0.51, 0.76	88
Managing relationships	0.57	0.46, 0.67	157
Social pressures (e.g., drinking, going out late, putting socializing before schoolwork)	0.58	0.45, 0.68	126
Meeting performance expectations	0.59	0.48, 0.68	170
Managing self-care and health (e.g., nutrition, exercise, taking time to rest or engage with hobbies)	0.58	0.47, 0.68	166
Financial concerns	0.70	0.61, 0.77	153
Concerns for the future (e.g., finding employment after graduation, hitting lifetime milestones)	0.62	0.51, 0.70	161
**PSS-10 score**	0.81	0.75, 0.86	171

Note: Non-parametric spearman’s rho correlation coefficients are presented, all of which were statistically significant at *p*<0.001. Abbreviations: Perceived Stress Scale 10-item version (PSS-10).

## Discussion

Mental health concerns continue to be an issue among post-secondary student populations, with recent research suggesting that above average stress, psychological distress, and symptoms consistent with mental illnesses have significantly increased over the past decade [7]. In response to the growing demand for mental health supports and in line with emergent broad-scale frameworks for supporting post-secondary mental health (i.e., the Okanagan Charter [44], the National Standard of Canada for Mental Health and Wellbeing for Post-secondary Students [45], many institutions have begun to move away from the more traditional medical model of treating symptoms of mental illness, instead adopting a more holistic approach to supporting overall student mental health and fostering a culture of wellbeing [46]. Mental health promotion and mental illness prevention strategies are critical upstream supports that aim to reduce the overall prevalence of languishing mental health and mental illness by providing students with the tools required to develop and improve resiliency and intervene prior to symptom development. While many institutions do indeed provide upstream mental health promotion supports to their students, national data collected from campus mental health service providers and institutional administrators in 2016 suggested that students seldom seek out these supports [47]. Furthermore, many respondents reported feeling that there was room for improvement in the services and supports currently provided [47]. A large part of improving upstream approaches designed to support students’ mental health and wellbeing involves the careful evaluation of the sources of student stress. Doing so will allow post-secondary institutions to effectively improve the targeting of their upstream supports, which may in turn alleviate the overwhelming burden currently placed on downstream treatment options.

The Post-Secondary Student Stressors Index (PSSI) is a useful tool for conducting a detailed evaluation of the sources of student stress, providing a rich, holistic assessment by evaluating nearly fifty potential stressors across five domains of student life. However, many institutions are already engaged in other lengthy survey tools designed to support the surveillance of student mental health and wellbeing, including the National College Health Assessment [6] and the Canadian Campus Wellbeing Survey [48]. While the PSSI adds a detailed assessment of student stress that is presently missing from these surveys, adding a 46-item instrument to these already lengthy questionnaires is not feasible. Doing so is likely to push respondents past their cognitive load capacity, which may potentially impact response rates as well as the quality of the data collected. The Brief-PSSI is a short, 14-item alternative that can be more easily added to these existing surveillance surveys without unduly increasing cognitive load. In addition, having been created based on a strategic grouping of stressors assessed the original PSSI, the Brief-PSSI continues to provide valuable insight into the most salient stressors experienced by students, assessed by both severity of stress elicited and frequency of occurrence. Though the tool evaluates fewer stressors overall, the five domains of student life assessed in the original instrument continue to be tapped, resulting in a high-level overview of general patterns of student stress that can provide institutions with guidance on how to improve the targeting of their upstream mental health supports. In this study, we were able to provide preliminary internal structure and relations to other variables evidence for validity for the Brief-PSSI, as well as strong test-retest reliability over a two-week period. Future research should continue to explore the validity of this novel instrument with larger, more varied samples.

### Limitations

As always, there are some limitations associated with this research. No formal content or response processes evidence were collected as part of this validation study for the Brief-PSSI. However, extensive content validation work was completed on the original, 46-item instrument and the brief version of the tool was created by strategically collapsing those stressors into smaller categories. The sample size used here was small, particularly the second sample used for test-retest reliability analyses. The majority of respondents were full-time, female undergraduate students between 21 and 24 years old, reporting a GPA in the A range. Overrepresentation in these categories may have impacted the overall results. Further, this sample is drawn from students attending a single university in Eastern Ontario, as well as during the Spring semester when many students are not enrolled. Additionally, the response rate of about 7% was low, though just below the expected range of response rates typically observed in survey-based research conducted among this population. As a result, possible selection bias may be present. Validation is an ongoing process. Future validation work conducted on the Brief-PSSI should examine evidence to support the validity of the tool among a larger, more varied sample comprised of students attending multiple institutions to ensure it is valid for use across different post-secondary contexts. Additionally, types of evidence for validity not presented in the current study should be examined (i.e., content, response processes). Finally, we did not have sufficient data to complete an assessment of measurement equivalence in this study. Future work should explore this method of validity testing as well.

## Supporting information

S1 Appendix Mean severity and frequency for stressors on Brief-PSSI across timepoints. (DOCX)

S2 Appendix Exploratory factor analysis conducted on PSSI and item reduction for development of Brief PSSI. (DOCX)

S3 Appendix Exploratory factor analysis of Brief PSSI. (DOCX)
