# Supplementary material for: Preliminary evidence for the validity of the Brief Post-Secondary Student Stressors Index (Brief-PSSI): A cross-sectional psychometric assessment
Source: PLoS One. 2024 Jan 19;19(1):e0297171. doi: 10.1371/journal.pone.0297171 (PMC10798508; doi:10.1371/journal.pone.0297171)
Supplement: S1 Appendix — (DOCX) [file pone.0297171.s001.docx]

**S1 Appendix**

**S1 Table.** Mean severity and frequency for stressors on Brief-PSSI across timepoints

|  | **Severity** | | | | **Frequency** | | | |
| --- | --- | --- | --- | --- | --- | --- | --- | --- |
|  | **T1** | | **T2** | | **T1** | | **T2** | |
| **Stressor** | **Mean** | **SD** | **Mean** | **SD** | **Mean** | **SD** | **Mean** | **SD** |
| 1. Examinations (i.e., midterms, finals) | 2.84 | 0.85 | 2.85 | 0.84 | 2.53 | 0.96 | 2.33 | 0.92 |
| 1. Managing my academic workload | 2.54 | 0.77 | 2.65 | 0.77 | 2.78 | 0.88 | 2.84 | 0.85 |
| 1. Managing my grades | 2.41 | 0.93 | 2.45 | 0.94 | 2.50 | 0.97 | 2.52 | 0.94 |
| 1. Lack of clarity in course instruction | 2.12 | 0.94 | 2.12 | 1.02 | 1.80 | 0.92 | 1.63 | 0.84 |
| 1. Interacting with faculty | 1.82 | 0.85 | 1.83 | 0.86 | 1.82 | 0.91 | 1.83 | 0.90 |
| 1. Adjusting to university life | 1.97 | 0.95 | 1.87 | 0.84 | 2.03 | 0.94 | 1.86 | 0.86 |
| 1. Pressure to succeed | 2.89 | 0.94 | 2.77 | 0.89 | 2.84 | 0.97 | 2.74 | 0.97 |
| 1. Discrimination (e.g., racism, sexism, etc.) | 1.83 | 0.83 | 1.81 | 0.91 | 1.68 | 0.86 | 1.56 | 0.86 |
| 1. Managing relationships | 2.18 | 0.90 | 2.09 | 0.88 | 2.19 | 0.96 | 2.28 | 0.95 |
| 1. Social pressures (e.g., drinking, going out late, putting socializing before schoolwork) | 1.83 | 0.92 | 1.70 | 0.79 | 1.83 | 0.91 | 1.80 | 0.89 |
| 1. Meeting performance expectations | 2.55 | 0.91 | 2.68 | 0.92 | 2.56 | 0.95 | 2.62 | 0.94 |
| 1. Managing self-care and health (e.g., nutrition, exercise, taking time to rest or engage with hobbies) | 2.51 | 1.00 | 2.48 | 0.94 | 2.62 | 1.00 | 2.77 | 0.97 |
| 1. Financial concerns | 2.53 | 1.05 | 2.47 | 1.10 | 2.49 | 1.05 | 2.50 | 1.11 |
| 1. Concerns for the future (e.g., finding employment after graduation, hitting lifetime milestones) | 2.83 | 1.03 | 2.84 | 1.03 | 2.71 | 1.05 | 2.61 | 1.02 |

Notes: Means were calculated excluding responses of 0 (“Didn’t Happen” or “Not Applicable”); SD = Standard Deviation; T1 = Timepoint 1, T2 = Timepoint 2.
