# Supplementary material for: Preliminary evidence for the validity of the Brief Post-Secondary Student Stressors Index (Brief-PSSI): A cross-sectional psychometric assessment
Source: PLoS One. 2024 Jan 19;19(1):e0297171. doi: 10.1371/journal.pone.0297171 (PMC10798508; doi:10.1371/journal.pone.0297171)
Supplement: S2 Appendix — (DOCX) [file pone.0297171.s002.docx]

**S2 Appendix**

**S2 Table.** Exploratory factor analysis conducted on PSSI and item reduction for development of Brief PSSI

| **Item** | **1** | **2** | **3** | **4** | **5** | **6** | **7** | **8** | **9** | **10** | **11** | **Brief-PSSI Item Development** |
| --- | --- | --- | --- | --- | --- | --- | --- | --- | --- | --- | --- | --- |
| *Academics* | | | | | | | | | | | | |
| 1 | .774 |  |  |  |  |  |  |  |  |  |  | 1. Examinations |
| 2 | .773 |  |  |  |  |  |  |  |  |  |  |  |
| 3 | .673 |  |  |  |  |  |  |  |  |  |  |  |
| 4 | .762 |  |  |  |  |  |  |  |  |  |  |  |
| 5 | .527 |  |  |  |  |  |  |  |  | .450 |  |  |
| 6 | .490 |  |  |  |  |  |  |  |  | .509 |  | 1. Managing academic workload |
| 7 |  |  |  |  |  | .354 |  |  |  | .501 |  |  |
| 8 | .450 |  |  |  |  |  |  |  | .320 |  |  | 1. Maintaining my grades |
| 9 | .453 |  |  |  |  |  |  |  | .420 | .355 |  |  |
| 10 |  |  |  | .837 |  |  |  |  |  |  |  |  |
| 11 |  |  |  | .551 |  |  |  |  |  |  |  |  |
| *Learning Environment* | | | | | | | | | | | | |
| 12 |  |  |  |  |  |  | .788 |  |  |  |  | 1. Lack of clarity in course instruction |
| 13 |  |  |  |  |  |  | .782 |  |  |  |  |  |
| 14 |  |  |  |  |  |  | .778 |  |  |  |  |  |
| 15 |  |  |  | .352 |  |  |  |  |  |  |  | 1. Interacting with faculty |
| 16 |  |  |  | .902 |  |  |  |  |  |  |  |  |
| 17 |  |  |  | .862 |  |  |  |  |  |  |  |  |
| *Campus Culture* | | | | | | | | | | | | |
| 18 |  |  |  |  |  | .668 |  |  |  |  |  | 1. Adjusting to post-secondary life |
| 19 |  |  |  |  |  | .740 |  |  |  |  |  |  |
| 20 |  | .586 |  |  |  |  |  |  |  |  |  |  |
| 21 |  | .535 |  |  |  | .514 |  |  |  |  |  | 1. Pressure to succeed |
| 22 |  | .582 |  |  |  | .479 |  |  |  |  |  |  |
| 23 |  | .597 |  |  |  | .327 |  |  |  |  |  |  |
| 24 |  |  |  |  |  |  |  |  |  |  | .807 | 1. Discrimination (racism, sexism, etc.) |
| 25 |  |  |  |  |  |  |  |  |  |  | .816 |  |

**Table A-1.** Continued

| **Item** | **1** | **2** | **3** | **4** | **5** | **6** | **7** | **8** | **9** | **10** | **11** | **Brief-PSSI Item** |
| --- | --- | --- | --- | --- | --- | --- | --- | --- | --- | --- | --- | --- |
| *Interpersonal* | | | | | | | | | | | | |
| 26 |  |  | .767 |  |  |  |  |  |  |  |  | 1. Managing relationships |
| 27 |  |  | .782 |  |  |  |  |  |  |  |  | 1. Social pressures (drinking, going out) |
| 28 |  | .324 | .567 |  |  |  |  |  |  |  |  |  |
| 29 |  |  | .744 |  |  |  |  |  |  |  |  |  |
| 30 |  | .306 | .501 |  |  |  |  |  |  | .344 |  |  |
| 31 |  | .738 | .319 |  |  |  |  |  |  |  |  | 1. Meeting performance expectations |
| 32 |  | .584 | .334 |  |  |  |  |  |  |  |  |  |
| 33 |  | .676 | .327 |  |  |  |  |  |  |  |  |  |
| 34 |  | .699 |  |  |  |  |  |  |  |  |  |  |
| *Personal* | | | | | | | | | | | | |
| 35 |  |  |  |  | .584 |  |  |  |  |  |  | 1. Managing self-care |
| 36 |  |  |  |  | .713 |  |  |  |  |  |  |  |
| 37 |  |  |  |  | .736 |  |  |  |  |  |  |  |
| 38 |  |  |  |  | .642 |  |  |  |  |  |  |  |
| 39 |  |  |  | .327 |  |  |  | .340 |  |  |  |  |
| 40 |  |  |  |  | .388 |  |  |  |  | .367 | .336 |  |
| 41 |  |  |  |  | .494 |  |  |  |  | .332 |  |  |
| 42 |  |  |  |  |  |  |  | .894 |  |  |  | 1. Financial concerns |
| 43 |  | .309 |  |  |  |  |  | .915 |  |  |  |  |
| 44 |  |  |  |  |  |  |  | .316 | .481 |  |  | 1. Concerns for the future |
| 45 |  |  |  |  |  |  |  |  | .747 |  |  |  |
| 46 |  |  |  |  |  |  |  |  | .428 |  |  |  |
| Eigenvalue | 12.1 | 3.3 | 2.6 | 1.9 | 1.8 | 1.6 | 1.4 | 1.4 | 1.2 | 1.2 | 1.1 |  |

**Note:** Principal components analysis with varimax rotation and Kaiser normalization. KMO statistics >0.7, Barlett’s test of sphericity p<0.001.
