# Supplementary material for: Preliminary evidence for the validity of the Brief Post-Secondary Student Stressors Index (Brief-PSSI): A cross-sectional psychometric assessment
Source: PLoS One. 2024 Jan 19;19(1):e0297171. doi: 10.1371/journal.pone.0297171 (PMC10798508; doi:10.1371/journal.pone.0297171)
Supplement: S3 Appendix — (DOCX) [file pone.0297171.s003.docx]

**S3 Appendix**

**S3 Table.** Exploratory factor analysis of Brief PSSI

|  | **Model 1**  **(n=128)** | **Model 2**  **(n=128)** | |
| --- | --- | --- | --- |
| *Items* | *Component 1*  *(Student Stressors)* | *Component 1*  *(Internal Stressors)* | *Component 2*  *(External Stressors)* |
| Examinations (i.e., midterms, finals) | 0.619 | 0.561 |  |
| Managing my academic workload | 0.694 | 0.636 |  |
| Managing my grades | 0.679 | 0.865 |  |
| Pressure to succeed | 0.695 | 0.539 |  |
| Meeting performance expectations | 0.741 | 0.654 |  |
| Concerns for the future (e.g., finding employment after graduation, hitting lifetime milestones) | 0.572 |  | 0.420 |
| Lack of clarity in course instruction | 0.453 |  | 0.381 |
| Interacting with faculty | 0.313 |  | 0.256 |
| Adjusting to university life | 0.588 |  | 0.449 |
| Discrimination (i.e., racism, sexism, etc.) | 0.391 |  | 0.366 |
| Managing relationships | 0.495 |  | 0.612 |
| Social pressures (e.g., drinking, going out late, putting socializing before schoolwork) | 0.494 |  | 0.576 |
| Managing self-care and health (e.g., nutrition, exercise, taking time to rest or engage with hobbies) | 0.586 |  | 0.503 |
| Financial concerns | 0.419 |  | 0.409 |
| *Model Fit Statistics* | | | |
| *R^2^* | 32.1% | 37.2% | |
| *X^2^* | 136.3 (*p*<0.001) | 96.6 (*p*<0.01) | |
| RMSA | 0.07 | 0.06 | |
| RMSEA (95% CI) | 0.07 (0.05, 0.09) | 0.06 (0.04, 0.09) | |
| TLI | 0.85 | 0.90 | |

**Note.** Component columns show factor loadings. For both models, KMO statisitc >0.7, Bartlett’s test of sphericity *p*<0.001. For model two, a varimax rotation with Kaiser normalization was used.
